# Surface‐Anchored Ticagrelor Gelatin Nanoparticles‐Platelets System for Enhanced Anti‐PD‐L1 Therapy Response and Boosted Chemotherapeutic Efficacy of Nanomedicines

**DOI:** 10.1002/EXP.20240084

**Published:** 2025-03-06

**Authors:** Qi Lu, Hao Ye, Jian Zhao, Xiaoyuan Fan, Kaiyuan Wang, Zeyu Han, Tian Liu, Lili Du, Jiaxuan Song, Helin Wang, Haotian Zhang, Zhonggui He, Jin Sun

**Affiliations:** ^1^ Department of Pharmaceutics Wuya College of Innovation Shenyang Pharmaceutical University Shenyang Liaoning P. R. China; ^2^ Institute of Pharmacy Harbin Medical University Harbin Heilongjiang P. R. China; ^3^ Multi‐Scale Robotics Lab (MSRL) Institute of Robotics & Intelligent Systems (IRIS) ETH Zurich Zurich Switzerland; ^4^ School of Life Science and Biopharmaceutics Shenyang Pharmaceutical University Shenyang Liaoning P. R. China; ^5^ Joint International Research Laboratory of Intelligent Drug Delivery Systems Ministry of Education Shenyang Pharmaceutical University Shenyang Liaoning P. R. China

**Keywords:** chemotherapy, immunotherapy, MMPs‐responsive release, platelet‐based delivery system, tumor‐associated platelets

## Abstract

The tumor microenvironment is characterized by immunosuppression and compromised intratumoral perfusion, which impairs the effectiveness of immune checkpoint inhibitors and nanomedicines. A significant challenge is the role of activated platelets, as they increase transfer‐mediated PD‐L1 expression from tumor cells and maintain the integrity of tumor vasculature. These platelets support tumor growth by stabilizing the vasculature and enabling immune evasion, as well as shielding tumor cells from immune detection. To address these platelet‐mediated negative antitumor effects, we have developed bioengineered platelets (PTNPs) with surface‐anchored ticagrelor‐loaded gelatin nanoparticles. This study utilizes the natural tendency of platelets to localize their activated counterparts into tumors. Upon binding to tumor‐associated activated platelets, the PTNPs release ticagrelor in response to the secreted matrix metalloproteinases by activated platelet, inhibiting further platelet activation. This reduction in platelet activation lessens platelet‐facilitated immunosuppression and diminishes the transferred‐PD‐L1 expression from cancer cells to platelets, thus enhancing the immune response of anti‐PD‐L1 therapy. Additionally, this strategy weakens the activated platelets’ contribution to tumor vascular integrity, improving the extravasation and chemotherapeutic efficacy of nanomedicines. Our findings highlight the crucial role of platelet activation in tumor biology and introduce PTNPs as an effective approach to disrupt tumor‐supporting platelet activities and enhance anticancer treatments efficacy.

## Graphical abstract

Ticagrelor nanoparticles modified platelets could adhere to tumor‐associated platelets by specific recruiting ability and responsively release ticagrelor under MMPs stimulation. The released ticagrelor could inhibit platelets activity, enhancing tumor treatment efficacy.

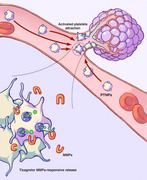

## 1 Introduction

Cancer persists as one of the foremost global health challenges, currently leading as a primary cause of mortality [1]. Traditional cancer treatments, including immunotherapy, chemotherapy, surgical intervention, and radiotherapy, have not achieved completely satisfactory outcomes in suppressing tumor progression [2a–c]. Recent evidences underscored the pivotal role of platelets in the tumor development [3a–c]. Notably, the rapid growth of tumors enables nanotherapeutics to penetrate tumor tissue through permeable vasculature, enhancing tumor‐targeted effects [4a,b]. However, this process weakens endothelial junctions, attracting resting platelets and initiating the coagulation cascade. The sequence of platelet activation, adhesion, and aggregation protects tumor vascular integrity, mitigates the enhanced permeability and retention (EPR) effect, and limits nanodrug accumulation at tumor sites, thereby reducing their therapeutic effect [5a–c].

Additionally, the resting platelets acquire PD‐L1 (programmed‐death ligand 1) from tumor cells through direct interaction and intracellularly store them in α‐granules. The stored PD‐L1 will be transferred from α‐granules to the surface with the platelet activation status, resulting in accumulated tumor‐derived PD‐L1 on the surface of platelets (tPD‐L1‐platelets) [6]. Similar to previously reported PD‐L1‐expressed exosomes [7], these tPD‐L1‐platelets could bind to PD‐1 (programmed‐cell‐death protein 1) on T lymphocytes, leading to the significant inhibition of the proliferation, activation, and cytotoxicity of CD8^+^ T cells, suppressing antitumor immunity systemically, and facilitating immune escape. The diminished efficacy of anti‐PD‐L1 (aPD‐L1) therapies might be attributed to the binding of these antibodies to numerous tPD‐L1‐platelets instead of directly targeting tumor cells. Moreover, the interactions between activated platelets and cancer cells promote tumor growth and survival, weakening the efficacy of therapeutic drugs. Consequently, increased platelet numbers and activity are associated with poor prognosis and decreased overall survival [8a,b]. Therefore, inhibiting platelets’ number and function emerges as a promising therapeutic strategy to alleviate tumor immunosuppression, disrupt tumor vascular integrity, and enhance the targeted delivery of chemotherapeutic nanomedicines [9a–d]. However, systemic depletion of platelets raises concerns about coagulation dysfunction and heightened systemic toxicity.

Here, platelet‐based delivery system is developed to address the problems above. As is known to all, platelets are accumulated and activated by the tumor inflammatory microenvironment [10a,b], which can recruit more circulating resting platelets through the cascade reaction [11a,b], therefore, platelet‐based delivery system can accurately anchor to the tumor‐associated platelets with reduced off‐target effects. In this study, we have developed a platelet‐based delivery system. Leveraging the natural accumulation and activation of platelets in the tumor inflammatory microenvironment, this system targeted tumor‐associated platelets with minimal off‐target effects. In addition, activated platelets secreted matrix metalloproteinases (MMPs) [12a,b], which specifically degraded gelatin nanoparticles [13a–c].

**SCHEME 1:**
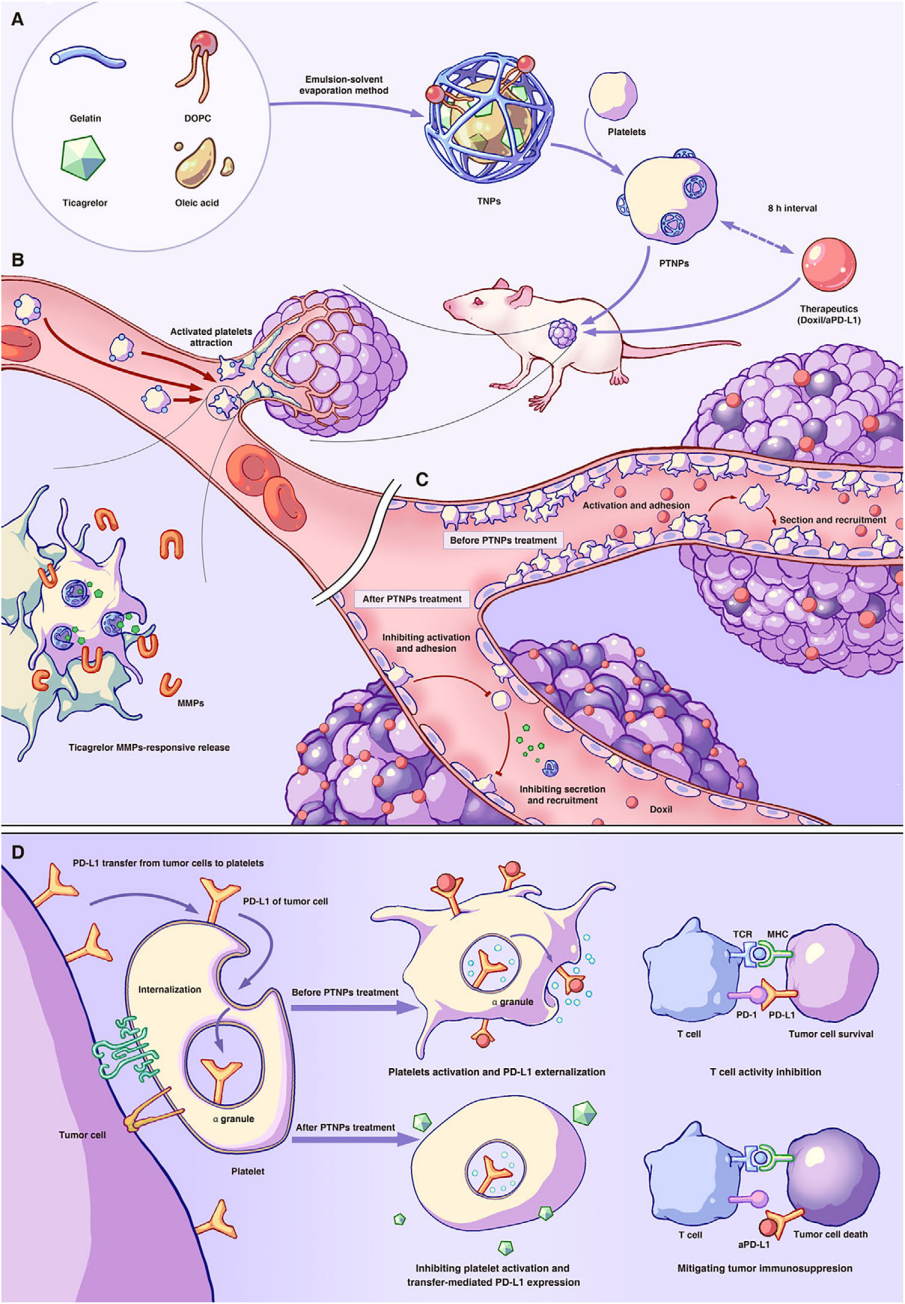
Schematic preparation of PTNPs and proposed action mechanism of PTNPs in vivo. (A) Schematic preparation of PTNPs. (B) PTNPs could adhere to tumor‐associated platelets by specific recruiting ability and responsively release ticagrelor under MMPs stimulation. (C) PTNPs inhibited platelets activity, leaded to the leaky tumor endothelial junctions, and promoted the penetration of anti‐tumor nanodrugs into tumor tissues. (D) PTNPs inhibited platelets activity, reduced transfer‐mediated PD‐L1 from tumor cells expression on platelets surface, and modulated tumor immunity.

In this study, we exploited the tumor‐targeting ability of platelets and the rapid MMPs‐responsive release capability of gelatin‐based particles to develop a novel delivery system as platelet activation inhibitors. This system aimed to minimize the systemic toxicity of anti‐platelet drugs and enhance their clinical applications. Specifically, we designed ticagrelor‐loaded gelatin nanoparticles (TNPs) and conjugated them to the surface of the resting platelets (PTNPs) (Scheme 1A). These PTNPs targeted the activated tumor‐associated platelets, where secreted MMPs to degrade TNPs, subsequently releasing ticagrelor (Scheme 1B). This process maintained the resting state of newly recruited platelets, disrupted tumor vascular barriers (Scheme 1C), and mitigated tPD‐L1‐platelets‐mediated tumor immunosuppression (Scheme 1D). In summary, PTNPs specifically targeted tumor sites and rapidly released ticagrelor to inhibit platelet activation. This approach holds promise for synergistic use with chemotherapeutic nanomedicines and immune checkpoint inhibitors, potentially enhancing long‐term efficacy in tumor therapy while reducing undesirable side effects, offering new hope for cancer patients.

## 2 Results

### 2.1 Preparation and Characterization of PTNPs

**FIGURE 1:**
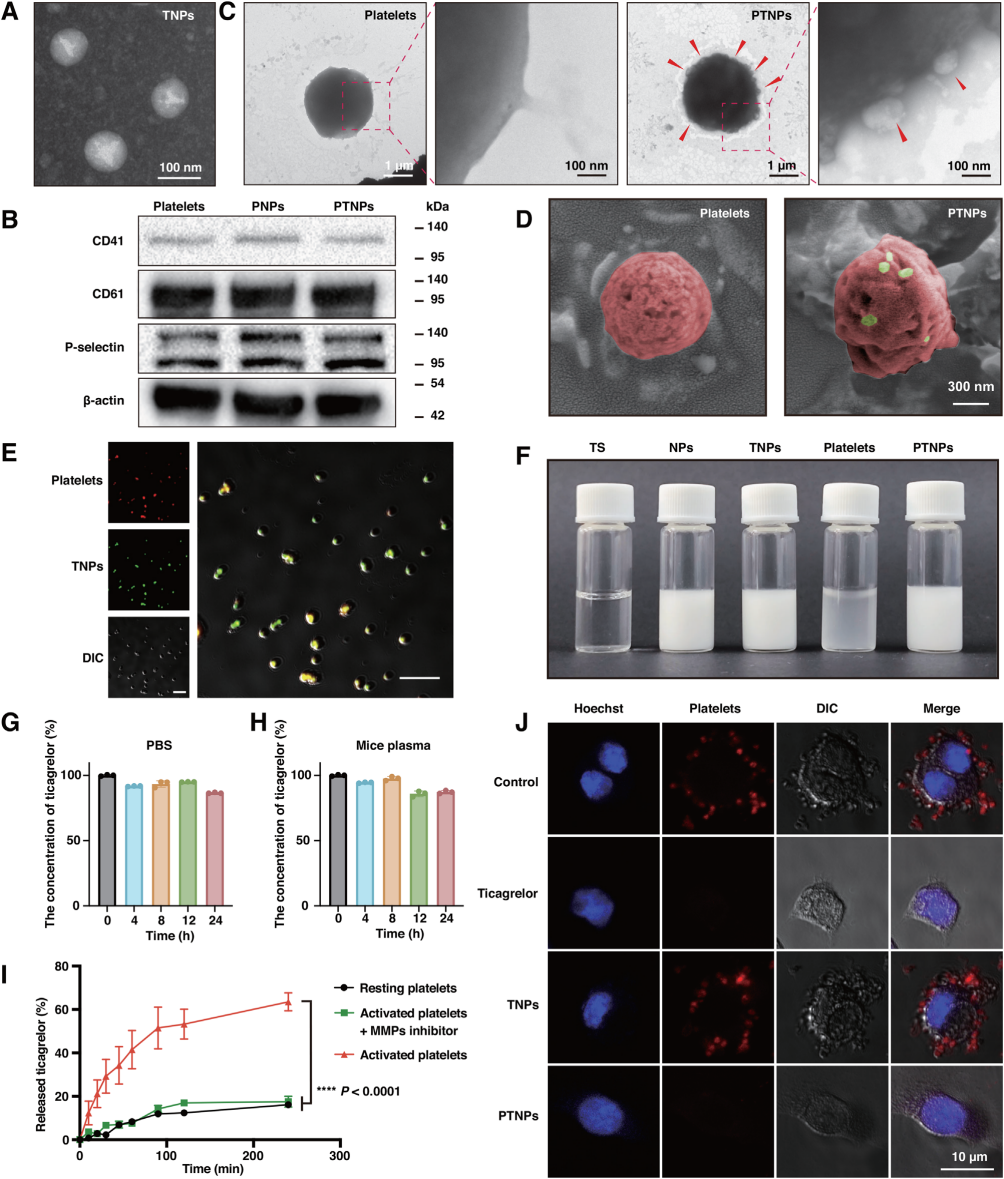
Characterization of PTNPs. (A) TEM images of TNPs. (B) Western blotting analysis of PNPs, and PTNPs for characteristic expression of platelet biomarkers CD41, CD61, P‐selectin, and β‐actin with untreated platelets as the control. (C) TEM images of platelets and PTNPs. The red arrows indicate TNPs. (D) Pseudocolor SEM images of platelets and PTNPs. Red and yellow mean platelets and TNPs. (E) CLSM images of PTNPs. Red: Platelets labeled with DiR. Green: TNPs labeled with Coumarin‐6. Scale bar: 10 µm. (F) Photograph of prepared samples. TS: ticagrelor solution. (G) The retention of TNPs on platelets surface in PBS with 1 µm PGE1 at room temperature for 24 h (*n* = 3). (H) The retention of TNPs on platelets surface in mice plasma at 37°C for 24 h (*n* = 3). (I) Release curves of free ticagrelor from TNPs in extracellular fluid (PBS, pH 7.4) of the activated or resting platelets with/without MMPs inhibitor (*n* = 3). (J) Adhesion of platelets to tumor cells with the treatment of different prepared samples. ****p < 0.0001 versus the control.

In accordance with the methodology described in Scheme 1A, ticagrelor‐loaded gelatin nanoparticles (TNPs) were synthesized using the emulsion solvent evaporation method, achieving a satisfactory drug loading efficiency of 92%. Transmission electron microscopy (TEM) images revealed homogeneous spherical TNPs with diameters averaging around 90 nm (Figure 1A). For the preparation of PTNPs (platelets with externally surface‐anchored TNPs), the fresh and resting platelets were isolated from mouse anti‐coagulated blood treated with prostaglandin E1 (PGE1). These platelets were then successfully conjugated with TNPs via a bifunctional maleimide covalent linker (sulfosuccinimidyl‐4‐(*N*‐maleimidomethyl)‐cyclohexane‐1‐carboxylate, Sulfo‐SMCC), achieving a coupling efficacy up to 14 nanoparticles per platelet (20.70 pg ticagrelor). To further investigate the effect of number of conjugated nanoparticles on the function of platelets, PNPs (platelets surface‐anchored blank nanoparticles with no ticagrelor) were prepared instead of PTNPs, for avoiding the interference of ticagrelor to the platelet carriers. It was observed that platelets’ adhesion ability was decreased as the number of conjugated nanoparticles increased. Specifically, platelets conjugated with 14 nanoparticles (PNPs‐14) exhibited only 50% of the adhesion capability compared to the untreated platelets. Conversely, PNPs‐10 (per platelet anchored with 10 nanoparticles) did not significantly impact their normal physiological functions (Figure S1, Supporting Information). Protein marker analysis including CD41, CD61, and P‐selectin, which are crucial in regulating platelet adhesion and aggregation, was performed through Western blotting. PTNPs‐10, with nearly 14.98 pg ticagrelor of per platelet, maintained expression of these vital membrane proteins without any detectable damage (Figure 1B), validating their use in the subsequent experiments.

Further analyses using TEM and scanning electron microscopy (SEM) confirmed the successful preparation of PTNPs, with TNPs (indicated by red arrows in Figure 1C and yellow in Figure 1D) conjugated on the surface of platelets (indicated by red in Figure 1D). Notably, there were no significant changes in the morphology of platelets, such as pseudopod formation, suggesting that TNPs modification did not induce platelet activation. This characteristic was advantageous for targeting tumor‐associated platelets accumulation and specific ticagrelor release. Resting platelets are essential for the desired therapeutic effect as they are recruited by tumor‐associated activated platelets and do not secrete matrix metalloproteinases (MMPs), thereby maintaining the integrity of TNPs with reduced non‐targeting toxicity. In addition, the modified TNPs showed almost no cytotoxicity to platelets in 48 h (Figure S2, Supporting Information). The successful binding of TNPs to platelets was further demonstrated through confocal laser scanning microscopy (CLSM, Figure 1E) and flow cytometry analysis (Figure S3, Supporting Information). The prepared PTNPs exhibited a uniform and milky‐white texture (Figure 1F). In addition, almost all of initially anchored TNPs were well‐retained on the surface of platelets during the storage of 24 h in PBS with PGE1 (Figure 1G and Figure S4A,B, Supporting Information). Similarly, exceeded 85% of TNPs were still anchored on platelet surface in 24 h upon the incubation of PTNPs in the mice plasma (Figure 1H and Figure S4C,D, Supporting Information), indicating that TNPs could steadily accompany platelets to the tumor site instead of off‐targeting effects.

Activated platelets, in contrast to resting ones, secrete MMPs [14], which could specifically degrade gelatin [13a]. The MMP‐responsiveness of TNPs was evaluated by co‐incubating them with supernatants from both resting and activated platelets. TNPs showed instability and rapid stratification when treated with activated platelet supernatant (Figure S5A, Supporting Information). Ticagrelor release was significantly more rapidly from TNPs treated with extracellular fluid at pH 7.4 from activated platelets (53.2% release at 2 h), compared to a negligible amount (12.3%) in the resting platelet group (Figure 1I), confirming the triggered release mechanism of TNPs. Additionally, the presence of the MMPs inhibitor significantly reduced ticagrelor release to levels comparable with the resting platelet group, indicating that MMPs predominantly facilitated ticagrelor release through gelatin hydrolysis. Ticagrelor release was also studied in an acidic tumor microenvironment, similar to its release behavior at pH 7.4 (Figure S5B, Supporting Information).

The activated platelets could adhere on the tumor cells surface [15a,b]. The interaction of tumor cells with PTNPs was studied by incubating tumor cells with different preparations for 2 h, followed by the introduction of DiR‐labeled resting platelets. The newly introduced platelets in both the free ticagrelor group and PTNPs group showed minimal adhesion, indicating rapid ticagrelor release from PTNPs, which in turn inhibited the activation of other platelets (Figure 1J and Figure S6, Supporting Information).

### 2.2 In Vivo Tumor‐Associated Platelets‐Targeting Ability of PTNPs

Tumor‐associated platelets, infiltrating the tumor microenvironment, became activated due to substances secreted by tumor cells, tumor‐expressed molecules, and exposure to endothelial cells [16a–c]. This activation amplified signals that recruited additional circulating platelets to tumor sites [11a,b]. To evaluate the recruitment potential of PTNPs by tumor‐associated platelets, we administered DiR‐labeled PTNPs intravenously through the tail vein in B16‐F10 tumor‐bearing mice. We then monitored the distribution of PTNPs at the tumor site over time.

**FIGURE 2:**
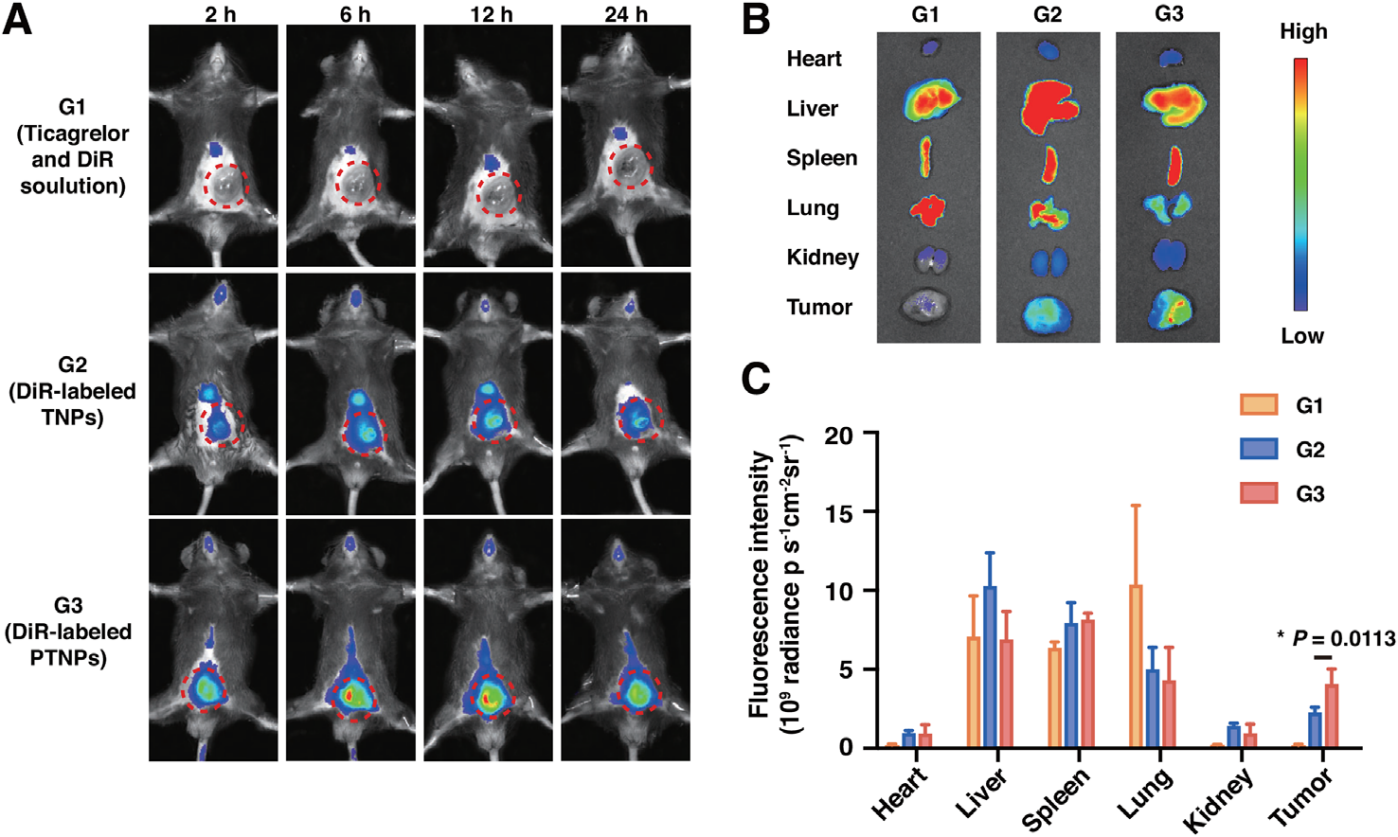
The targeting ability of tumor‐associated platelets of PTNPs. (A) In vivo fluorescence images of B16‐F10 tumor‐bearing mice at 2, 6, 12, and 24 h after intravenously administrated with free ticagrelor and DiR solution (G1), DiR‐labeled TNPs (G2), and DiR‐labeled PTNPs (G3), respectively. Tumor positions were circled by red dotted rings. (B) Ex vivo fluorescence images of major tissues at 24 h after injection. (C) Semi‐quantitative analysis of fluorescence intensity of major tissues (*n* = 3). *p < 0.05 versus the control.

Comparatively, DiR‐labeled TNPs, along with free ticagrelor and DiR solution, predominantly showed high fluorescence intensity in the liver and lung, respectively, with only the minority present in the tumor (Figure 2). In stark contrast, tumors treated with DiR‐labeled PTNPs exhibited bright fluorescence signals, suggesting that the targeted accumulation of PTNPs at tumor sites was facilitated by the recruitment activity of tumor‐associated platelets.

Furthermore, the peak accumulation of PTNPs in tumors was observed at 6 h post‐intravenous injection. Consequently, other therapeutic agents were administered 8 h following PTNPs infusion. This timing was strategically chosen to maximize PTNPs distribution and ticagrelor release within the tumor microenvironment, aiming to enhance the combined therapeutic effects in the subsequent experiments.

### 2.3 PTNPs Reduce Transfer‐Mediated PD‐L1 Expression on Platelet Surfaces and Modulate aPD‐L1‐Triggered Anti‐Tumor Immune Response

Currently, tumor immunotherapy has been widely studied, such as inducing immunogenic cell death [17], inhibiting tumor energy metabolism [18], and activating the stimulator of interferon genes pathway [19a,b], among which the most classic is still inhibiting immune checkpoints [20a,b]. The immune checkpoints, PD‐L1 protein, known for its immunosuppressive role, could be transferred from the surface of tumor cells into α‐granules of platelets through cell interactions, subsequently appearing on the platelet surface upon α‐granule secretion by activated platelets [6]. This transfer of tumor‐derived PD‐L1 to platelets could deplete progressive T‐cells, facilitating tumor immune evasion. Notably, PD‐L1 was overexpressed on the surface of B16‐F10 cells (Figure S7, Supporting Information).

**FIGURE 3:**
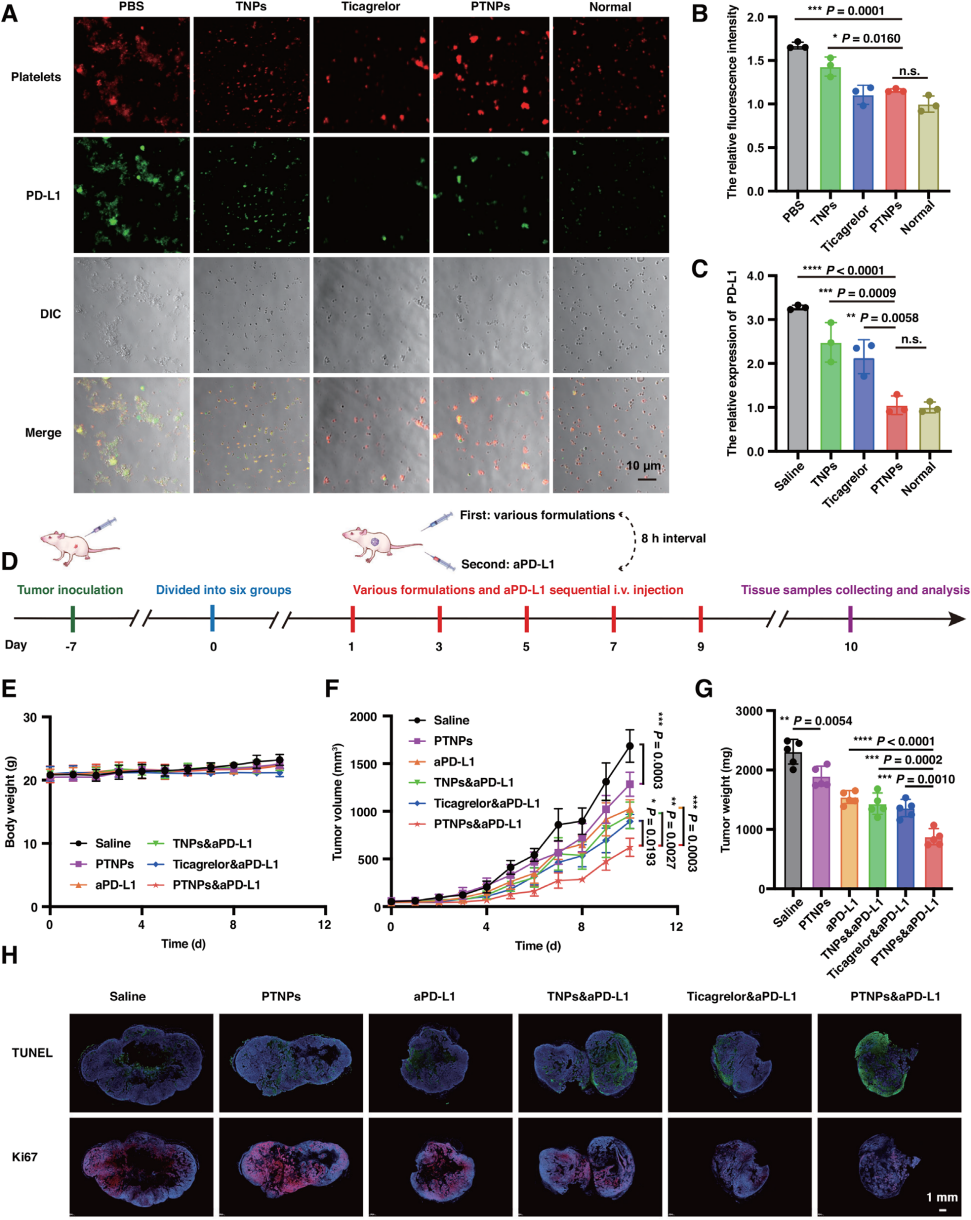
PTNPs reduced transfer‐mediated PD‐L1 surface expression on platelets from tumor cells and enhanced tumor immunotherapeutic efficacy. (A) Representative immunofluorescence staining of PD‐L1 on platelets surface. The platelets from healthy mice were co‐incubated with PD‐L1 over‐expressed tumor cells with different prepared samples. Red: DiR labeled platelets. Green: FITC‐labeled PD‐L1. (B) Flow cytometric analyses of the relative fluorescence intensity of platelets treated by tumor cells in vitro (*n* = 3). (C) ELISA analyses of PD‐L1 expression on the surface of platelets in tumor‐bearing mice in vivo (*n* = 3). (D) Diagrammatic representation of in vivo study schedule for aPD‐L1 therapy in B16‐F10‐tumor‐bearing mice. (E) Body weight curves of mice (*n* = 5). (F) Tumor volume curves of mice (*n* = 5). (G) Tumor weight of mice on day 10 (*n* = 5). (H) Representative images of the dissected tumor tissues of different groups in TUNEL and Ki67 staining assays. Blue: DAPI. Green: apoptotic area. Red: Ki67 positive sites. *p < 0.05, **p < 0.01, ***p < 0.001, ****p < 0.0001, and n.s., no significant difference versus the control.

To assess the impact of PTNPs on PD‐L1 expression on platelets, we incubated PTNPs with B16‐F10 tumor cells for 2 h. Subsequently, new DiR‐labeled platelets were added and incubated for an additional hour, after which PD‐L1 expression on the surface of these newly added platelets was observed. Platelets treated with PBS and TNPs exhibited higher green fluorescence intensity (Figure 3A). Flow cytometry analysis revealed no significant difference in fluorescence intensities between PTNPs‐treated platelets and normal ones, suggesting that PTNPs could reduce transfer‐mediated tumor PD‐L1 expression on platelet surfaces by inhibiting the activation of these cells (Figure 3B).

These findings were further validated in vivo. B16‐F10 tumor‐bearing mice were treated with saline, TNPs, ticagrelor, and PTNPs, and then platelets were extracted from the blood post 8 h. Platelets from normal healthy mice served as a control. ELISA quantitative analysis indicated a 3.2‐fold reduction in PD‐L1 expression on the tumor‐associated platelets in mice treated with PTNPs compared to saline control (Figure 3C), suggesting that PTNPs, in combination with aPD‐L1, could potentially overcome the low response rate of immunotherapy and enhance its effectiveness.

The in vivo anti‐tumor activity of aPD‐L1 was further evaluated in B16‐F10 tumor‐bearing C57BL/6j mice (Figure 3D). 7 days post‐inoculation, when tumor sizes reached approximately 50 mm^3^, mice were divided into six groups and received intravenous administrations of saline, PTNPs, aPD‐L1, TNPs&aPD‐L1, ticagrelor&aPD‐L1, and PTNPs&aPD‐L1 every other day (Ticagrelor: 10 mg kg^−1^, aPD‐L1: 2 mg kg^−1^, with an 8‐h interval between injections). During the 10‐day period, mice treated with ticagrelor&aPD‐L1 remained the body weight, whereas mice in other groups steadily gained weight (Figure 3E). The PTNPs&aPD‐L1 group demonstrated significantly lower tumor growth rates and tumor volumes compared to other groups, highlighting the potent anti‐tumor effect of the PTNPs and aPD‐L1 combination (Figure 3F,G).

Specifically, PTNPs inhibited the activation of tumor‐associated platelets, reduced PD‐L1 expression on their surface, and alleviated the tumor's immunosuppressive microenvironment. aPD‐L1 could combine with PD‐L1 of tumor cells, preventing T cells from inactivation and tumor cells from evading attack. Moreover, PTNPs alone also exhibited anti‐tumor effects without cytotoxicity (Figure S8, Supporting Information), suggesting that PTNPs could enhance autoimmunity by regulating platelet status. TUNEL, Ki67, and H&E stained tissue slices further demonstrated significant inhibition of tumor differentiation with PTNPs&aPD‐L1 treatment (Figure 3H and Figure S9, Supporting Information).

### 2.4 Effective Immunotherapy for Tumor Inhibition

**FIGURE 4:**
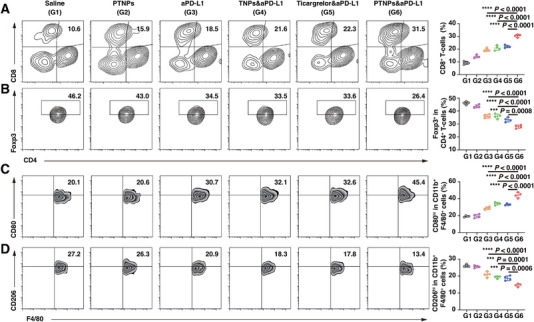
PTNGs modulated the aPD‐L1‐triggered anti‐tumor immune response. (A) Representative flow cytometric examination images as well as relative quantification of CD8^+^ T cells (*n* = 4). (B) Representative flow cytometric examination images as well as relative quantification of CD4^+^ Foxp3^+^ T cells (*n* = 4). (C) Representative flow cytometric examination images as well as relative quantification of CD80^hi^F4/80^+^CD11b^+^CD45^+^ macrophages (M1‐like) (*n* = 4). (D) Representative flow cytometric examination images as well as relative quantification of CD206^hi^F4/80^+^CD11b^+^CD45^+^ macrophages (M2‐like) (*n* = 4). ***p < 0.001, and ****p < 0.0001 versus the control.

To substantiate the immunotherapeutic efficacy of our approach, we analyzed tumor infiltrating lymphocytes (TILs) and tumor‐associated macrophages (TAMs) extracted from tumors on day 10 using flow cytometry. The CD8^+^ effector T cells exhibited a modest increase with both PTNPs (G2) and aPD‐L1 (G3) treatment, indicating that strategies targeting tPD‐L1‐platelets or utilizing aPD‐L1 could moderately activate the T cell‐mediated immune response (Figure 4A). Remarkably, the combination of PTNPs and aPD‐L1 (PTNPs&aPD‐L1, G6) elicited a more potent immune response, evidenced by a 2.31‐fold increase in tumor‐infiltrating cytotoxic T lymphocytes and a 0.39‐fold decrease in suppressor T cells (Figure 4A,B). Furthermore, compared to control groups, there was an observable increase in proinflammatory M1‐like TAMs and a decrease in anti‐inflammatory M2‐like TAMs following PTNPs&aPD‐L1 treatment, confirming the promotion of a robust immune response (Figure 4C,D).

These findings elucidated the superior anti‐tumor effects which we observed with PTNPs&aPD‐L1 treatment (Figure 3G). PTNPs effectively targeted tumor‐associated platelets and mitigated immune suppression by reducing tPD‐L1‐platelet numbers. The synergy with aPD‐L1 revitalized TILs and facilitated the phenotypic transformation of M2 TAMs into M1 TAMs, culminating in direct tumor eradication.

Furthermore, the spleen, a critical organ in the immune system known to be enlarged under immunocompromised conditions [21a–c], was also examined. The spleens harvested from saline and aPD‐L1 groups exhibited splenomegaly, whereas those from mice treated with PTNPs&aPD‐L1 maintained a normal size (Figure S10, Supporting Information). This observation further corroborated the exceptional immunotherapeutic effect of the PTNPs&aPD‐L1 combination.

### 2.5 PTNPs Modulate Tumor Retention of Doxil and Enhance Chemotherapeutic Efficacy

**FIGURE 5:**
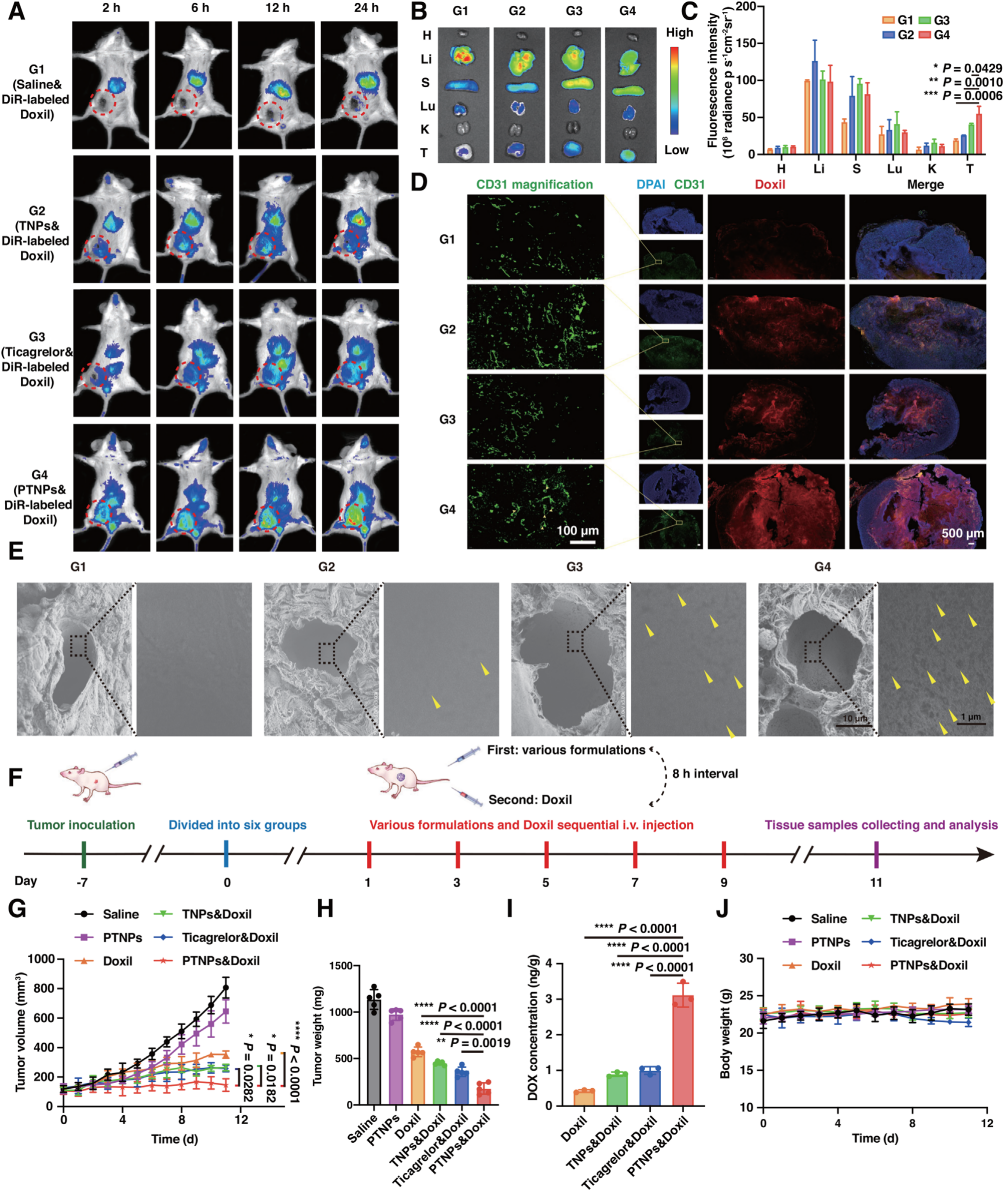
PTNPs modulation on the tumor retention of Doxil and boosting chemotherapeutic efficacy. (A) In vivo fluorescence images of 4T1 tumor‐bearing mice with different formulations treatments at 2, 6, 12, and 24 h after intravenously administrated with DiR‐labeled Doxil. Tumor positions were circled by red dotted rings. G1: Saline& DiR‐labeled Doxil. G2: TNPs& DiR‐labeled Doxil. G3: Ticagrelor& DiR‐labeled Doxil. G4: PTNPs& DiR‐labeled Doxil. (B) Ex vivo fluorescence images of major tissues at 24 h after injection. H: heart. Li: liver. S: spleen. Lu: lung. K: kidney. T: tumor. (C) Semi‐quantitative analysis of fluorescence intensity of major tissues (*n* = 3). (D) Doxil distribution of tumor tissues at 24 h after injection. Red: Doxil. Blue: DAPI. Green: CD31. The endothelial breaches were labeled by yellow arrows. (E) Scanning electron micrographs of blood vessels in 4T1 tumors. Yellow arrows indicated the widened breaches on the blood vessels. (F) Diagrammatic representation of in vivo study schedule for Doxil therapy in 4T1‐tumor‐bearing mice. (G) Tumor volume curves of mice (*n* = 5). (H) Tumor weight of mice on day 11 (*n* = 5). (I) DOX concentration of tumors (*n* = 3). (J) Body weight curves of mice (*n* = 5). *p < 0.05, **p < 0.01, ***p < 0.001, and ****p < 0.0001 versus the control.

The defective openings in tumor vasculature provided a pathway for nanoparticles to selectively enter the tumor interstitium from leaky blood vessels and remain retained for extended periods. However, the critical role of activated platelets in maintaining tumor vessel integrity could limit the perfusion of nanomedicines through this mechanism. To evaluate the capability of PTNPs in enhancing the tumor retention of nanomedicines, DiR‐labeled Doxil, serving as a model drug, was intravenously injected 8 h after various sample infusions. As depicted in Figure 5A–C, the fluorescence signal at the tumor site in PTNPs‐treated mice gradually increased over 24 h, whereas in the TNPs or ticagrelor groups, it peaked at 12 h post‐injection. This indicated prolonged Doxil retention at tumor sites, likely due to the strong recruitment and aggregation abilities of platelets. PTNPs continuously targeted and released ticagrelor to inhibit platelet activation at the tumor site, maintaining specifically leaky tumor endothelial junctions, which aided in the accumulation of Doxil. Immunofluorescence analysis of Doxil in tumors (Figure 5D) revealed negligible red fluorescence in the Doxil group, attributed to tight endothelial junctions restricting Doxil extravasation into the tumor stroma. In contrast, significant Doxil penetration was observed in other groups (TNPs, ticagrelor, and PTNPs), with PTNPs being the most effective. In addition, the tumor blood vessels were detected through SEM. Compared with the saline control group, the blood vessels emerged the apparent and widened breaks following PTNPs treatment (Figure 5E). These results supported that PTNPs reduced the endothelial integrity through platelet inhibiting, and then facilitated the enhanced permeability and retention effect of Doxil at tumor sites, which provided the excellent theoretical basis for the good efficacy of the combination of PTNPs and Doxil.

To ascertain if PTNPs pretreatment could amplify chemotherapeutic efficacy, the anti‐tumor activity of Doxil was further evaluated in 4T1 tumor‐bearing Balb/c mice (Figure 5F). Tumor‐bearing mice received various ticagrelor‐containing formulations (ticagrelor: 10 mg kg^−1^), followed by Doxil injection (2 mg kg^−1^, equivalent Dox dosage) 8 h later. Mice treated with PTNPs&Doxil exhibited pronounced inhibition of tumor growth, whereas PTNPs alone marginally delayed tumor growth (Figure 5G,H), suggesting Doxil's role in tumor eradication and PTNPs’ primary function in enhancing Doxil retention at the tumor site. Tumor concentrations of Doxil, measured via fluorescence spectrometry on Day 11, were 2.06‐, 2.40‐, and 5.49‐fold higher in the PTNPs&Doxil group compared to ticagrelor&Doxil, TNPs&Doxil, and Doxil groups, respectively (Figure 5I). This finding underscored PTNPs’ strong ability to promote Doxil penetration by inhibiting platelet activation.

Moreover, systemic toxicity assessments revealed slight weight loss in the ticagrelor&Doxil‐treated mice (Figure 5J), possibly due to systemic hematological toxicity. These mice also suffered from pulmonary hemorrhage, as evidenced in Figure S11, Supporting Information, while other groups exhibited no significant bleeding or morphological changes. Hepatorenal toxicity evaluations showed slight increases in alanine aminotransferase (ALT), aspartate aminotransferase (AST), and blood urea nitrogen (BUN) levels in the ticagrelor&Doxil group (Figure S12, Supporting Information), indicating potential hepatic and renal toxicity. Notably, the PTNPs&Doxil treatment maintained a favorable safety profile, attributable to the targeted platelet delivery and MMPs‐responsive gelatin nanoparticle release.

## 3 Discussion

Platelets are known to significantly contribute to tumor progression through mechanisms such as extracellular vesicle production, transforming growth factor‐β secretion, and the formation of platelet‐cloaked circulating tumor cell aggregates [22]. Importantly, these physiological processes are contingent upon platelet activation [23]. Furthermore, activated platelets play a pivotal role in maintaining vascular homeostasis, which can effectively obstruct the diffusion of nanodrugs into solid tumors [24]. Emerging evidences indicated that activated platelets may carry tumor cell‐derived immunosuppressive protein PD‐L1, potentially inhibiting cytotoxic T cell activity and facilitating immune evasion by tumor cells [6]. Thus, mitigating the adverse effects of activated platelets is crucial in the antitumor therapy.

In this study, we leveraged the tumor‐associated platelet‐targeting capability of platelets and the MMPs‐sensitive cleavage ability of gelatin nanoparticles to develop bioengineered platelets with surface‐anchored ticagrelor‐loaded nanoparticles (PTNPs) aimed at modulating tumor immunity and enhancing chemotherapeutic efficacy of nanomedicines. PTNPs interacted with tumor‐associated platelets to create a ticagrelor‐rich tumor microenvironment, effectively impeding platelet recruitment, activation, adhesion, and aggregation. On the one hand, PTNPs mitigated the immunosuppressive microenvironment, stimulated T cell activation, and boosted the effectiveness of immunotherapy. On another, they inhibited vascular endothelial repair, increased nanomedicine leakage, and amplified anti‐tumor effects. Thanks to their activated platelet‐specific targeting, PTNPs were likely to exhibit minimal toxicity to normal tissues and significantly to reduce the risk of systemic bleeding. In conclusion, PTNPs held considerable promise in the treatment of multiple tumor types. They offered synergistic treatment possibilities when combined with various nanomedicines and immune checkpoint inhibitors, opening new avenues for advanced cancer therapies.

## 4 Methods

### 4.1 Materials

Ticagrelor was purchased from Macklin Reagent (Shanghai, China). 1,2‐dioleoyl‐sn‐glycero‐3‐phosphocholine (DOPC) was obtained from AVT Pharmaceutical Technology Co., Ltd. (Shanghai, China). Oleic acid was supplied by Aladdin Biochemical Technology Co. Ltd. (Shanghai, China). Gelatin, sulfosuccinimidyl‐4‐(*N*‐maleimidomethyl)‐cyclohexane‐1‐carboxylate (Sulfo‐SMCC), 1,1′‐dioctadecyl‐3,3,3′,3′‐tetramethylindotricarbocyanine iodide (DiR), Dulbecco's modified eagle medium (DMEM), Roswell Park Memorial Institute (RPMI‐1640), TBST, sensitive ECL luminescence reagent, Fetal bovine serum (FBS), and 3‐(4,5‐dimethylthiazol‐2yl)‐2,5‐diphenylterazolium bromide (MTT) were purchased from Dalian Meilun Biotech Co., Ltd. (Dalian, China). Cell culture dishes were purchased from NEST Biotechnology Co., Ltd, (Wuxi, China). Hoechst 33342 and Coumarin 6 (C6) supplied by Beijing Solarbio Science and Technology Co., Ltd. (Beijing, China). The anti‐PD‐L1 antibody was obtained from Biolegend (cat.no. 124329, Clone:10F.9G2). Doxorubicin liposome (Doxil) was obtained from Cspc Ouyi Pharmaceutical Co., Ltd. (Shijiazhuang, China).

### 4.2 Preparation of PTNPs

As the previous reports [13, 25], gelatin nanoparticles entrapping ticagrelor (TNPs) were prepared by the oil‐in‐water emulsion solvent evaporation method with minor modification. In brief, 10 mg ticagrelor, 5 mg DOPC, and 100 µL oleic acid were solubilized in 1 mL dichloromethane. The organic phase was added dropwise into 5% gelatin and 1% PVA solution and sonicated for 3 min at 450 w in the iced bath. The obtained emulsion was rotary evaporated to remove organic solvent and mixed with Sulfo‐SMCC for 30 min. The resultant TNPs were washed with equal volumes of PBS in the centrifugal filter device (molecular weight cut‐off = 100 kDa) between each step for three times. The same treatments were used to prepare blank nanoparticles without ticagrelor.

Fresh platelets were purified as previously reported [26a,b]. Briefly, anticoagulated mouse whole blood was centrifuged at 200 g for 10 min to obtain platelet‐rich plasma, which was suspended in the acid‐citrate‐dextrose solution (ACD) with prostaglandin E1 (PGE1, 1 µm, MedChemExpress, Monouth Junction, NJ, USA) and centrifuged at 800 g for another 10 min at room temperature. The obtained pure platelets were incubated with Sulfo‐SMCC‐modified TNPs in ACD with PGE1 for 30 min to obtain TNPs‐anchored platelets (PTNPs). PTNPs were centrifuged, washed, and finally resuspended in saline. The same treatments were used to prepare blank nanoparticles‐anchored platelets (PNPs) without ticagrelor. Noted that the fresh PTNPs were prepared for all experiments.

### 4.3 Characterization of PTNPs

To examine the concentrations of ticagrelor encapsulated in the NPs, TNPs were centrifuged in the centrifugal filter device (molecular weight cut‐off = 100 kDa) to remove unencapsulated ticagrelor. The upper TNPs and uncentrifuged TNPs were extracted and dissolved in acetonitrile to analyze the concentration by high performance liquid chromatography (HPLC) with Waters detector (*λ* = 242 nm). Column: C_18_ (250 mm, 4.6 mm); Mobile phase: acetonitrile/water = 60: 40 (v/v); Column temperature: 30°C; Flow rate: 1.0 mL min^−1^.

The concentrations of ticagrelor coupled on the surface of platelets were also calculated by HPLC. Briefly, PTNPs were centrifuged at 800 g for 10 min at room temperature. The precipitate and uncentrifuged PTNPs were treated with acetonitrile and centrifuged at 13,000 g for 5 min.

The morphology of the samples was observed by SEM (scanning electron microscopy, ZEISS GeminiSEM300. Germany) and TEM (transmission electron microscopy, Hitachi, HT7700, Japan). For the fluorescence signal, DiR‐labeled platelets and C6‐labeled TNPs were obtained by CLSM (confocal laser scanning microscope, TCS SP2/AOBS, LEICA, Germany).

For the retention of TNPs in PTNPs during storage, PTNPs were incubated in PBS with PGE1 (1 µm) at room temperature and in plasma at 37°C with shaking, respectively. At specific time point, 1 mL of the incubated mixture was extracted and centrifuged at 800 g for 10 min at room temperature. The precipitate was vortexed with acetonitrile for 5 min and then centrifuged at 13,000 g for another 5 min. The concentration of ticagrelor was analyze by HPLC as above description. For fluorescence detection, C6‐labeled PTNPs (platelets anchoring C6‐labeled TNPs) were prepared and analyzed by CLSM.

### 4.4 TNPs‐Treated Platelets Viability

The fresh platelets were inoculated in 96‐well plates (4 × 10^6^ platelets/well) with 200 µL TNPs with various concentrations for 48 h. TNPs solution was replaced by 20 µL MTT solution after centrifuging at 800 g for 10 min. 4 h later, 150 µL DMSO was added into 96‐well plates and the absorbance was detected at 570 nm by the microplate reader.

### 4.5 Expression of Characteristic Markers

The total cellular proteins of platelets, PNPs (blank nanoparticles anchored platelets without ticagrelor), and PTNPs, were extracted by protein extraction kit (RIPA:PMSF = 100:1, Beijing Dingguo Changsheng biotechnology Co., Ltd. China) at 4°C for 15 min. All samples were incubated with 0.5 U mL^−1^ thrombin for 30 min before extracting. The extracted proteins were boiled for 5 min with the protein concentration of 1 mg mL^−1^. And then, 20 µL samples were separated by 10% SDS‐PAGE gel electrophoresis with 80 V, 20 min, and 150 V, 30 min, sequentially. The separated proteins were subsequently transferred onto PVDF (polyvinylidene fluoride) membranes with 250 mA, 45 min, and blocked in 5% skim milk for 2 h at room temperature. Next, the membranes were treated with the primary antibody against CD61 (ABclonal, A19073), CD41 (ABclonal, A11490), P‐selectin (ABclonal, A12512), and β‐actin (ABclonal, AC006) at a 1:1000 dilution overnight at 4°C and then treated with the second antibody (Goat anti‐rabbit IgG (H+L), ABclonal, AS070) at a dilution of 1:10,000 for 1 h at room temperature. The membranes were treated by TBST for three times to remove the unbound antibodies and then treated with sensitive ECL luminescence reagent to fluoresce in specific region. The western blot images were obtained by the gel imaging system (Bio‐Rad, Singaporean) and analyzed by Image Lab software (6.0.1).

For western blotting analysis of B16‐F10 tumor cells, the method was similar to before, and the primary antibody against PD‐L1 (ABclonal A19135) was used.

### 4.6 In Vitro Ticagrelor Release

The resting or activated platelet supernatant solution (pH 6.5 and 7.4) containing 0.5% DMSO (v/v), with or without MMPs inhibitor GM6001, was used as the release medium. At selected time points, the dialysate was extracted and replaced with an equivalent volume of fresh‐release media. The released ticagrelor was determined by HPLC as above description.

### 4.7 Cell Culture

Murine melanoma cells (B16‐F10) and mouse breast cancer cells (4T1) were bought from the Cell Bank of Type Culture Collection of Chinese Academy of Sciences (Shanghai, China). B16‐F10 and 4T1 were cultured in high‐glucose DMEM and RPMI‐1640 medium, respectively, with 10% FBS, streptomycin sulfate (100 µg mL^−1^), and penicillin (100 units mL^−1^). All cells were grown in a 37°C, 5% CO_2_ cell incubator.

### 4.8 Adhesion of PNPs to B16‐F10 Cells

B16‐F10 cells (1 × 10^4^ cells) were seeded in confocal dishes at 37°C. After 24 h adherence, the confocal dishes were then moved to 4°C refrigerators for 20 min, following DiR‐labeled PNPs added into the dishes for another 1 h at 4°C. The nuclei were counterstained by Hoechst 33342. Then, the adhesion was observed by CLSM.

### 4.9 Adhesion of Platelets to B16‐F10 Cells

B16‐F10 cells (1 × 10^4^ cells) were seeded in confocal dishes at 37°C. TNPs, ticagrelor, and PTNPs were incubated with tumor cells, respectively, with ticagrelor concentrations of 0.5 mg mL^−1^. 2 h later, DiR‐labeled fresh and resting platelets were added into the dishes for 1 h at 4°C. The nuclei were counterstained by Hoechst 33342. Then, the adhesion was observed by CLSM.

### 4.10 Cytotoxicity Assay

To determine the cell viability of PTNPs, B16‐F10 cells (2 × 10^3^ cells per well) were seeded in 96‐well plate. After overnight cell growth, various concentrations PTNPs were added and incubated for 48 h. The MTT assay was applied, and the OD values were determined at 570 nm with the micro‐plate reader.

### 4.11 Animals

Balb/c mice (standard Balb/c strain without specific substrain differentiation, female) and C57BL mice (6j substrain, male) were supplied by the Animal Center of Shenyang Pharmaceutical University. All mice were post‐weaning and pre‐adult, with 6–8 weeks at the start of the experiment. In addition, all mice were assessed for general health and behavior prior to the commencement of the study. No animals exhibited signs of illness or abnormal behavior, ensuring uniformity in the study cohort.

### 4.12 In Vivo Tumor Models

For B16‐F10 tumor‐bearing mice model, B16‐F10 cells (1 × 10^6^) were subcutaneously injected into the right flank of C57BL/6j mice. When the tumor volumes reached 50 mm^3^, the B16‐F10 tumor‐bearing mice were randomly divided into six groups and injected intravenously with saline, PTNPs, aPD‐L1, TNPs&aPD‐L1, ticagrelor&aPD‐L1, and PTNPs&aPD‐L1 (equivalent ticagrelor dose: 10 mg kg^−1^, aPD‐L1 dose: 2 mg kg^−1^), respectively, once every 2 days. Note that the ticagrelor‐containing formulations and aPD‐L1 were injected at a time interval of 8 h. Tumor diameter and body weight were measured every day. On the 10th day, mice were sacrificed, and primary organs and tumors were obtained, washed with cold saline, weighted, and fixed with 4% formaldehyde.

Similar to B16‐F10 model, 4T1 cells (1 × 10^6^) were subcutaneously injected into the left fat pad of Balb/c mice to establish 4T1 tumor‐bearing mice model. When the tumor volumes reached 120 mm^3^, saline, PTNPs, Doxil, TNPs&Doxil, ticagrelor&Doxil, and PTNPs&Doxil (equivalent ticagrelor dose: 10 mg kg^−1^, equivalent Dox dose: 2 mg kg^−1^), respectively, were injected intravenously once every 2 days. And the mice were sacrificed on the 11th day.

### 4.13 In Vivo Biodistribution of PTNPs

The gelatin nanoparticles entrapping ticagrelor and DiR (DiR‐labeled‐TNPs), and DiR‐labeled PTNPs (platelets anchoring DiR‐labeled‐TNPs) were prepared. B16‐F10 tumor‐bearing C57BL/6j mice model was established, and the mice were injected intravenously with free ticagrelor and DiR solution, DiR‐labeled TNPs, and DiR‐labeled PTNPs, respectively (equivalent ticagrelor dose: 10 mg kg^−1^, equivalent DiR dose: 1 mg kg^−1^). At specific time points, in vivo imaging system (IVIS) spectrum small‐animal imaging system was used to obtain in vivo NIRF images with a noninvasive optical at an excitation wavelength of 748 nm. After 24 h post‐injection, the tumors and major organs from euthanized mice were harvested for analysis with the same IVIS system and settings.

### 4.14 PD‐L1‐Expression Level on Platelets Surface

B16‐F10 cells (1 × 10^4^ cells per well) were seeded in 12‐well plate for 24 h. TNPs, ticagrelor, and PTNPs were incubated with tumor cells, respectively, with ticagrelor concentrations of 0.5 mg mL^−1^. 2 h later, DiR‐labeled fresh and resting platelets were added into the dishes for 1 h. Then, the platelet suspension was centrifuged at 800 g for 10 min and the collected platelets were treated with Alexa Fluor 488 Anti‐PD‐L1 antibody (Abcam, ab281751). The transfer‐mediated PD‐L1 expression on platelet surface was observed by CLSM and analyzed quantitatively by flow cytometry.

To evaluate PD‐L1‐expression level on platelet surface in vivo, B16‐F10 tumor‐bearing C57BL/6j mice were used. Briefly, saline, TNPs, ticagrelor, and PTNPs were injected intravenously with ticagrelor at an equivalent concentration of 10 mg kg^−1^, respectively. 8 h later, the blood (10 µL) was obtained from the tail. Platelets from normal healthy mice were used as the control. The membranes of platelets were extracted by Cytoplasmic protein extraction kit (Dalian Meilun Biotech Co., Ltd., China). The PD‐L1 was measured by ELISA kit (Enzyme‐Linked Immunosorbent Assay, Cloud‐Clone Corp, L220105868).

### 4.15 In Vivo Biodistribution of Doxil

Similar to in vivo biodistribution of PTNPs part, 4T1 tumor‐bearing Balb/c mice model was established and the mice were injected intravenously with saline, TNPs, ticagrelor, and PTNPs (equivalent ticagrelor dose: 10 mg kg^−1^), respectively. 8 h later, DiR‐labeled Doxil (equivalent DiR dose: 1 mg kg^−1^) was injected intravenously.

### 4.16 Flow Cytometry

Tumors extracted from the mice were chopped into small sections and homogenized in cold staining buffer with the presence of the digestive enzyme (Beijing Solarbio Science and Technology Co., Ltd. China) to yield single‐cell suspensions. Staining of the cells with fluorescence‐labeled antibodies, CD80 (Biolegend, cat. no. 104722, clone 16‐10A1), CD45 (Biolegend, cat. no. 103108, clone 30‐F11), CD206 (Biolegend, cat. no. 141716, clone C068C2), CD11b (Biolegend, cat. no. 101208, clone M1/70) and F4/80 (Biolegend, cat. no. 123116, clone BM8) was performed following the protocols of manufacturer.

For T cells analysis, the cell mixtures were treated with lymphocyte separation kits (Beijing Solarbio Science and Technology Co., Ltd. China) to purify T cells. After that the cells were stained with fluorescence‐labeled antibodies, CD3 (Biolegend, cat. no.100204, clone 17A2), CD4 (Biolegend, cat. no. 100432, clone GK1.5), CD8 (Biolegend, cat. no. 100712, clone 53–6.7), Foxp3 (Biolegend, cat. no. 126404, clone MF‐14), as the outlined instructions of manufacturer.

All of the stained cells were measured by flow cytometer (BD FACSCalibur) and evaluated by FlowJo software (version 10.4.0) with the presentation as percentage‐based cells in the flow cytometry analysis images.

### 4.17 Statistics

All results are presented as mean ± s.d. Tukey post‐hoc tests and one‐way analysis of variance (ANOVA) were used. All data were analyzed by GraphPad Prism 8. Statistical significance was considered at ^*^ *p* < 0.05, ^**^ *p* < 0.01, ^***^ *p* < 0.001 and ^****^ *p* < 0.0001.

## Author Contributions

Qi Lu, Hao Ye, and Jin Sun conceived the project. Qi Lu, Jian Zhao, Xiaoyuan Fan, Zeyu Han, Tian Liu, Lili Du, Jiaxuan Song, and Helin Wang performed the experiments. Qi Lu, Hao Ye, Kaiyuan Wang, and Jin Sun analyzed the results. Zhonggui He and Haotian Zhang provided useful suggestions for this work. Qi Lu, Hao Ye, and Jin Sun wrote the manuscript.

## Ethics Statement

All animals were supplied by the Animal Center of Shenyang Pharmaceutical University. The execution of all animal experiments meticulously adhered to the Guidelines for the Care and Use of Laboratory Animals. All animal procedures and experiments were in conformity under the guidelines provided by the Institutional Animal Ethical Care Committee (IAECC) of Shenyang Pharmaceutical University, with the approved protocol number for the laboratory is SYPU‐IACUC‐C2022‐1‐14‐102.

## Conflicts of Interest

The authors declare no conflicts of interest.

## Supporting information



Supporting Information

## Data Availability

All data associated with this study are present in the main text or Supporting Information. Additional data are available upon request to the authors.
