# Supplementary material for: Surface‐Anchored Ticagrelor Gelatin Nanoparticles‐Platelets System for Enhanced Anti‐PD‐L1 Therapy Response and Boosted Chemotherapeutic Efficacy of Nanomedicines
Source: Exploration (Beijing). 2025 Mar 6;5(3):20240084. doi: 10.1002/EXP.20240084 (PMC12199361; doi:10.1002/EXP.20240084)
Supplement: Supplementary file 1 — Supporting Information [file EXP2-5-20240084-s001.docx]

**Supporting information**

**Surface-Anchored Ticagrelor Gelatin Nanoparticles-Platelets System for Enhanced Anti-PD-L1 Therapy Response and Boosted Chemotherapeutic Efficacy of Nanomedicines**

*Qi Lu ^a,b,#^, Hao Ye ^c,#^, Jian Zhao ^a^, Xiaoyuan Fan ^a^, Kaiyuan Wang ^a^, Zeyu Han ^a^, Tian Liu ^a^, Lili Du ^a^, Jiaxuan Song ^a^, Helin Wang ^a^, Haotian Zhang ^d^, Zhonggui He ^a,e^, Jin Sun ^a,e*^*

*^a^* Department of Pharmaceutics, Wuya College of Innovation, Shenyang Pharmaceutical University, 103 Wenhua Road, Shenyang Liaoning, 110016, P. R. China.

*^b^* Institute of Pharmacy, Harbin Medical University, 157 Baojian Road, Harbin Heilongjiang, 150081, P. R. China.

*^c^* Multi-Scale Robotics Lab (MSRL), Institute of Robotics & Intelligent Systems (IRIS), ETH Zurich, Zurich 8092, Switzerland.

*^d^* School of Life Science and Biopharmaceutics, Shenyang Pharmaceutical University, 103 Wenhua Road, Shenyang Liaoning, 110016, P. R. China.

^e^ Joint International Research Laboratory of Intelligent Drug Delivery Systems, Ministry of Education, Shenyang Pharmaceutical University, Shenyang Liaoning, 110016, P. R. China.

^#^ These authors contributed equally to this work.

^*^ Corresponding author: Prof. Jin Sun PhD. E-mail: sunjin@syphu.edu.cn.


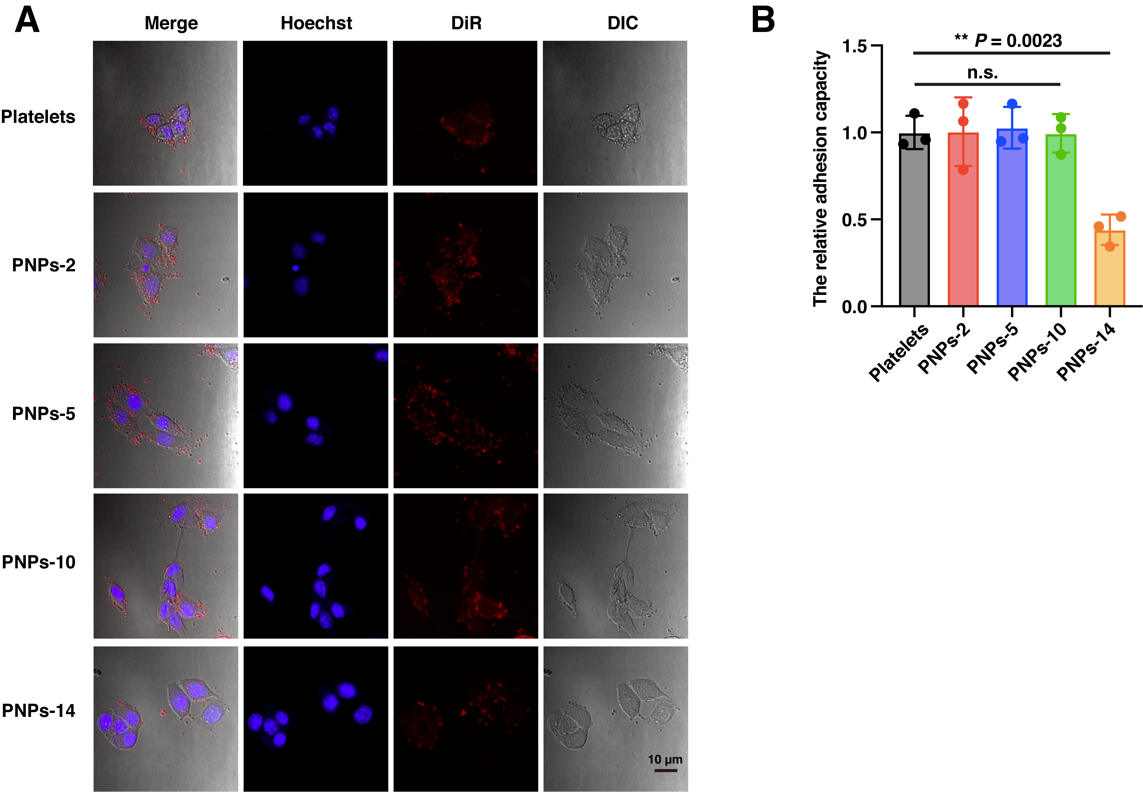


Figure S1. The adhesion of tumor cells and PNPs with different ratios of platelets to NPs. (A) The images observed by confocal laser scanning microscope. (B) Quantitative analysis of adhesion capacity (n = 3). ** *P* < 0.01 and n.s., no significant difference versus the control.


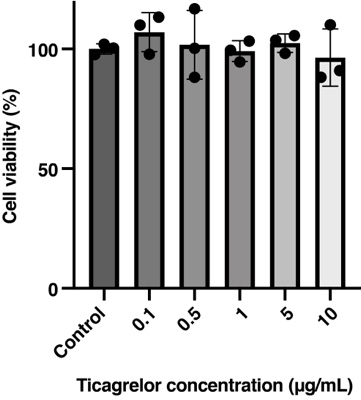


Figure S2. *In vitro* cytotoxicity of 48 h after TNPs treatment against platelets (n = 3).


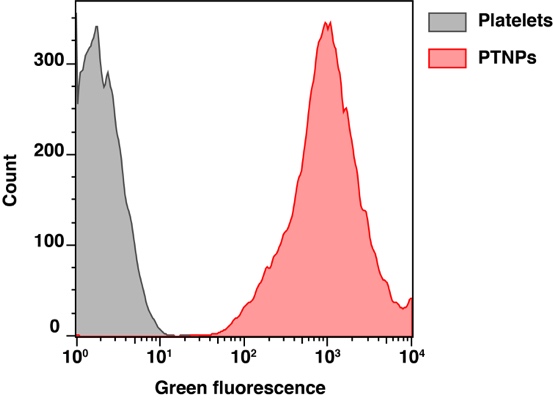


Figure S3. The flow cytometry analysis of untreated platelets and PTNPs. The TNPs were labeled with Coumarin-6.


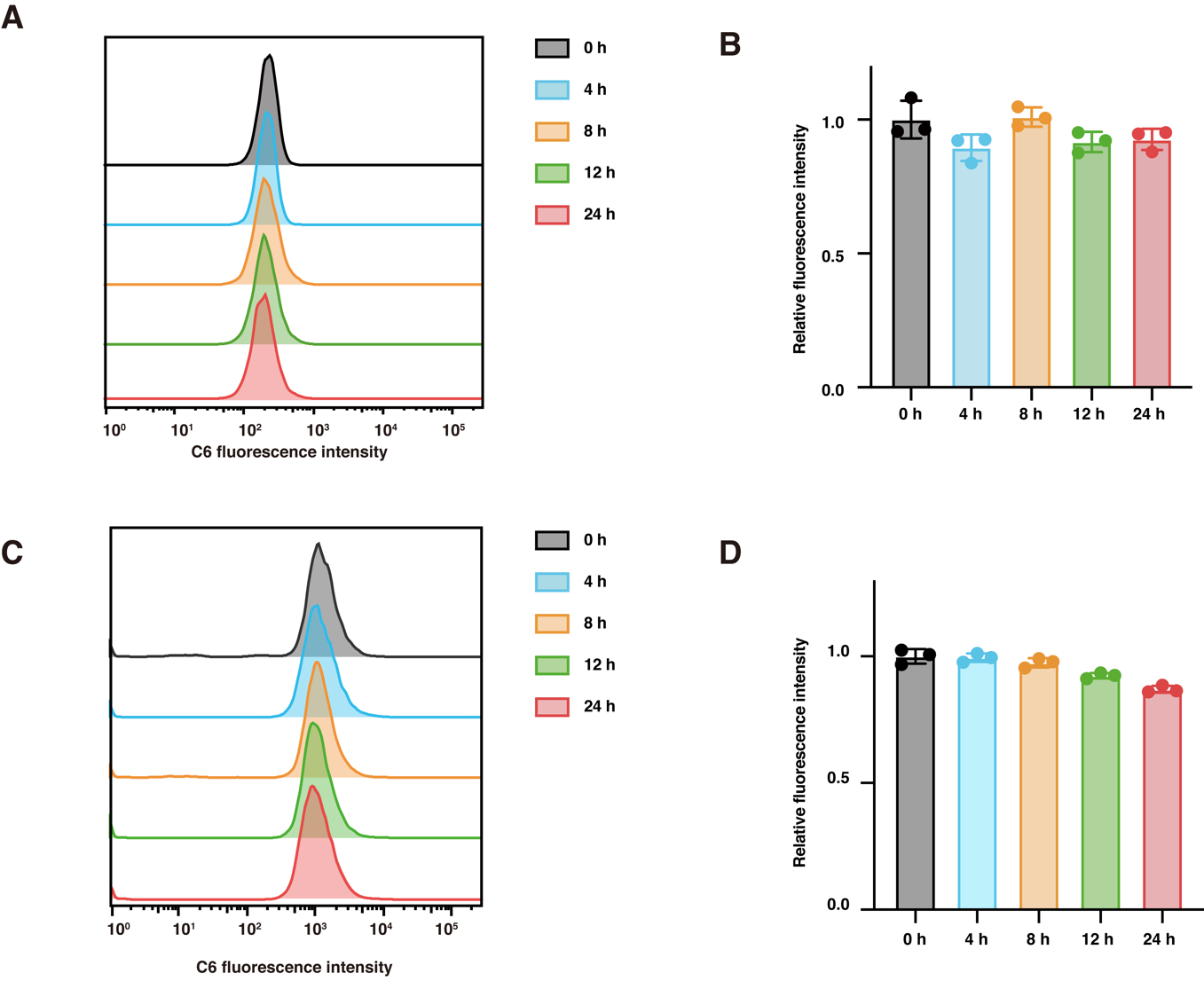


Figure S4. (A) Flow cytometry analysis of the retention of TNPs in PTNPs at room temperature for 24 h in PBS with PGE1 (1 µM). The TNPs were labeled with Coumarin-6 (C6). (B) Results for flow cytometry analysis (n = 3). (C)Flow cytometry analysis of the retention of TNPs on platelet surface in mice plasma at 37℃ for 24 h. (D) The relative fluorescence intensity for flow cytometry analysis (n = 3).


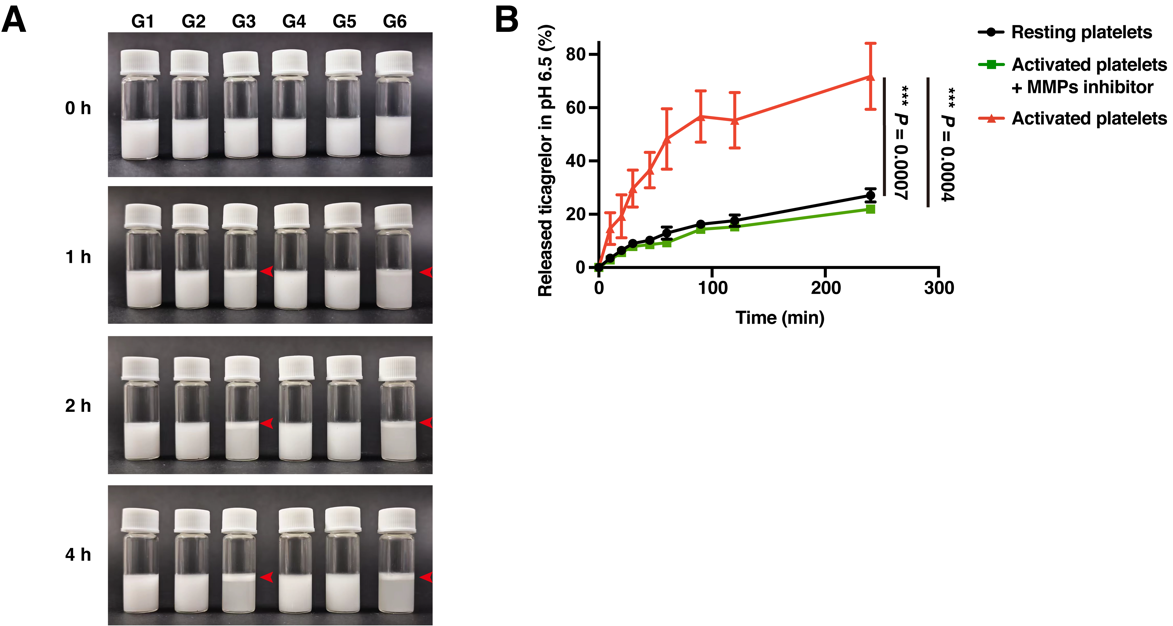


Figure S5. Release of free ticagrelor from TNPs. (A) Photograph of TNPs with different treatments at 0, 1, 2, and 4 h. G1: Extracellular fluid of resting platelets (pH 7.4). G2: Extracellular fluid of activated platelets with MMPs inhibitor (pH 7.4). G3: Extracellular fluid of activated platelets (pH 7.4). G4: Extracellular fluid of resting platelets (pH 6.5). G5: Extracellular fluid of activated platelets with MMPs inhibitor (pH 6.5). G6: Extracellular fluid of activated platelets (pH 6.5). (B) Release curves of free ticagrelor from TNPs in extracellular fluid (PBS, pH 6.5) of the activated or resting platelets with/without MMPs inhibitor (n = 3). *** *P* < 0.001 versus the control.


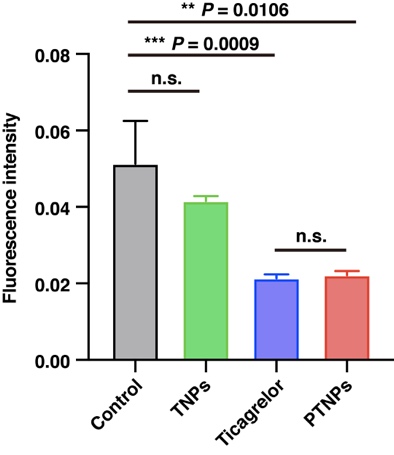


Figure S6. Semiquantitative analysis of adhesion with ImageJ software (n = 3). ** *P* < 0.01, *** *P* < 0.001, and n.s., no significant difference versus the control.


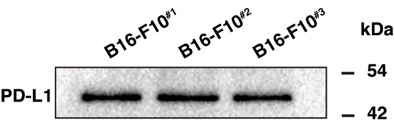


Figure S7. Western blotting analysis of B16-F10 tumor cells for expression PD-L1.


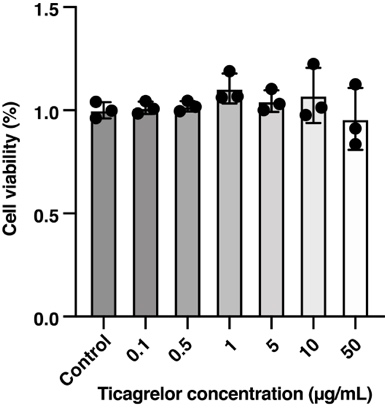


Figure S8. *In vitro* cytotoxicity of 48 h after PTNPs treatment against B16-F10 tumor cells (n = 3).


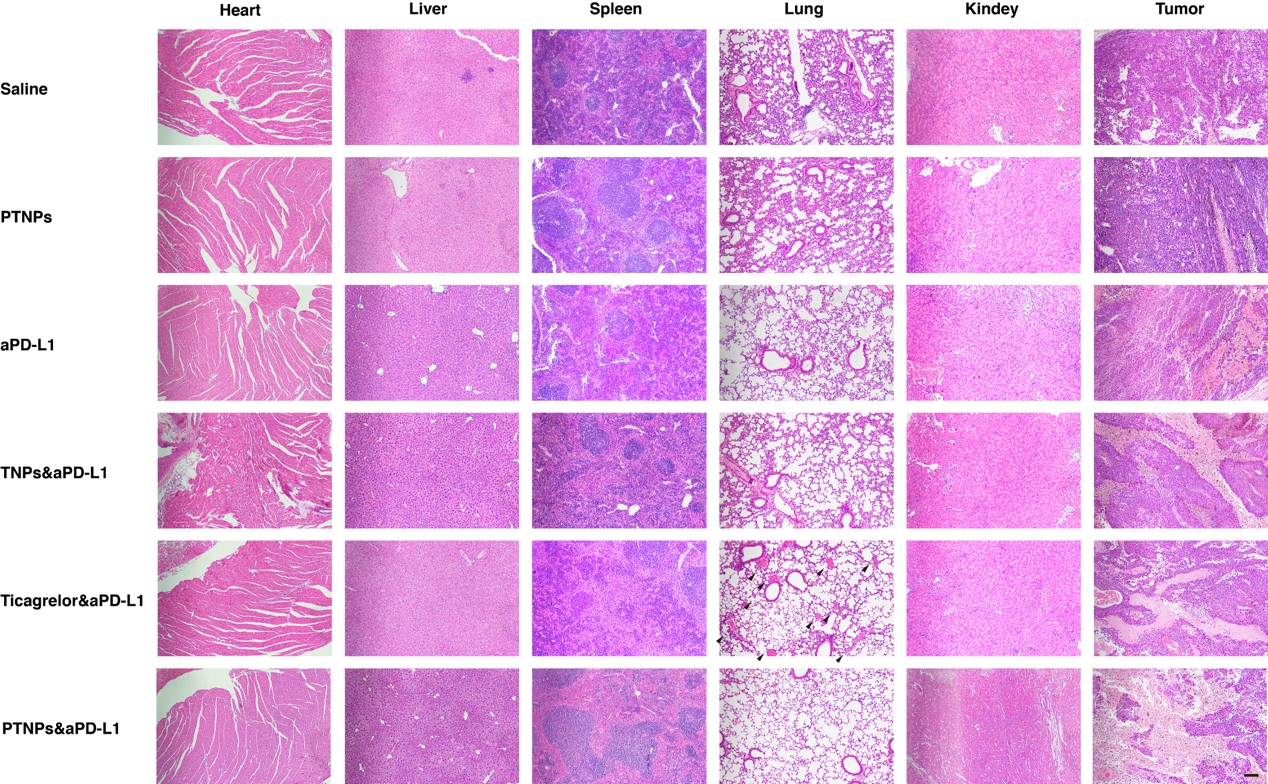


Figure S9. H&E staining images for heart, liver, spleen, lung, kidney, brain, and tumor slices after different treatments. Black arrows represent blood clots. Scale bar: 100 µm.


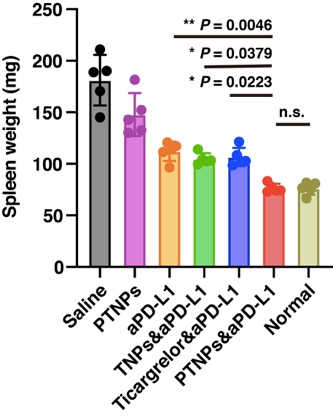


Figure S10. Weight of representative spleens in different groups (n = 5). * *P* < 0.05, ** *P* < 0.01, and n.s., no significant difference versus the control.


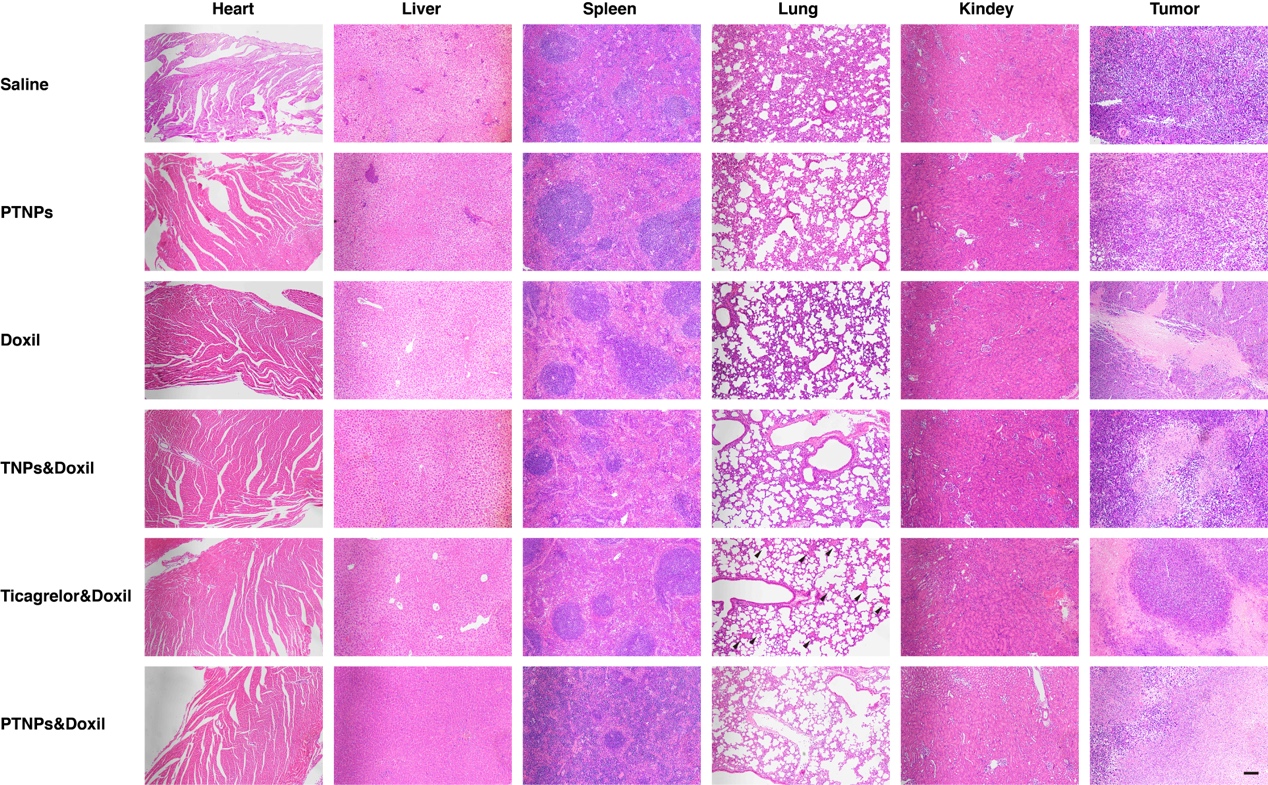


Figure S11. H&E staining images for heart, liver, spleen, lung, kidney, brain, and tumor slices after different treatments. Black arrows represent blood clots. Scale bar: 100 µm.


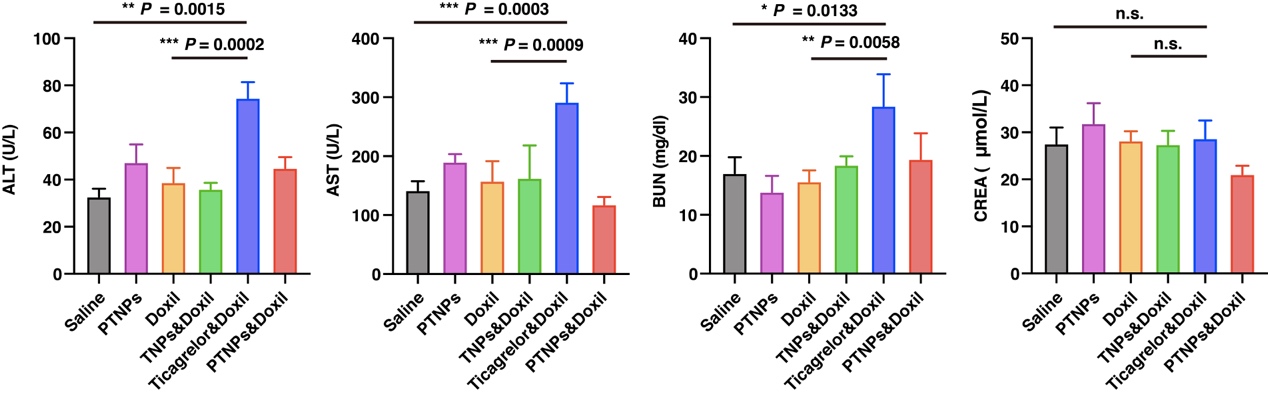


Figure S12. The hematological parameters of mice with different treatments (n = 3). Alanine aminotransferase (ALT). Aspartate aminotransferase (AST). Blood urea nitrogen (BUN). Creatinine (CREA). * *P* < 0.05, ** *P* < 0.01, *** *P* < 0.001, and n.s., no significant difference versus the control.
